# Is early-onset colorectal cancer an evolving pandemic? Real-world data from a tertiary cancer center

**DOI:** 10.1093/oncolo/oyae239

**Published:** 2024-10-02

**Authors:** Angelos Angelakas, Thekla Christodoulou, Konstantinos Kamposioras, Jorge Barriuso, Michael Braun, Jurjees Hasan, Kalena Marti, Vivek Misra, Saifee Mullamitha, Mark Saunders, Natalie Cook

**Affiliations:** Department of Medical Oncology, The Christie NHS Foundation Trust, Manchester M20 4BX, United Kingdom; Department of Medical Oncology, The Christie NHS Foundation Trust, Manchester M20 4BX, United Kingdom; Department of Medical Oncology, The Christie NHS Foundation Trust, Manchester M20 4BX, United Kingdom; Department of Medical Oncology, The Christie NHS Foundation Trust, Manchester M20 4BX, United Kingdom; Department of Medical Oncology, The Christie NHS Foundation Trust, Manchester M20 4BX, United Kingdom; Department of Medical Oncology, The Christie NHS Foundation Trust, Manchester M20 4BX, United Kingdom; Department of Medical Oncology, The Christie NHS Foundation Trust, Manchester M20 4BX, United Kingdom; Department of Clinical Oncology, The Christie NHS Foundation Trust, Manchester M20 4BX, United Kingdom; Department of Medical Oncology, The Christie NHS Foundation Trust, Manchester M20 4BX, United Kingdom; Department of Clinical Oncology, The Christie NHS Foundation Trust, Manchester M20 4BX, United Kingdom; The Christie NHS Foundation Trust and Division of Cancer Sciences, Faculty of Biology, Medicine and Health, University of Manchester, Manchester M20 4BX, United Kingdom

**Keywords:** early onset, colorectal cancer, prognosis, real-world data

## Abstract

**Background:**

Early onset Colorectal Cancer (EOCRC), defined as those diagnosed under the age of 50, has been increasing rapidly since 1970. UK data on EOCRC are currently limited and better understanding of the condition is needed.

**Materials and Methods:**

A single-center retrospective study of patients with EOCRC treated over 9 years (2013-2021) at a large UK cancer center was performed. Clinicopathological features, risk factors, molecular drivers, treatment, and survival were analyzed.

**Results:**

In total, 203 patients were included. A significant increase in cases was reported from 2018-2019 (*n* = 33) to 2020-2021 (*n* = 118). Sporadic EOCRC accounted for 70% of cases and left-sided tumors represented 70.9% (*n* = 144). Median duration of symptoms was 3 months, while 52.7% of the patients had de-novo metastatic disease. Progression-free survival after first-line chemotherapy was 6 months (95% CI, 4.85-7.15) and median overall survival (OS) was 38 months (95% CI, 32.86-43.14). In the advanced setting, left-sided primary tumors were associated with a median OS benefit of 14 months over right-sided primaries (28 vs 14 months, *P* = .009). Finally, primary tumor resection was associated with median OS benefit of 21 months compared with in situ tumors (38 vs 17 months, *P* < .001).

**Conclusions:**

The incidence of EOCRC is increasing, and survival outcomes remain modest. Raising public awareness and lowering the age for colorectal cancer screening are directions that could improve EOCRC clinical outcomes. There is also a need for large prospective studies to improve the understanding of the nature of EOCRC and the best therapeutic approaches.

**Implications for practice**

Early onset colorectal cancer (EOCRC) is an increasing problem worldwide. In our study, sporadic EOCRC and left-sided primary tumors accounted for 70% of cases. More than 50% of patients presented with metastatic disease, despite most presenting with a less than 6-month history of symptoms. Even in patients with metastatic presentation, primary tumor resection was linked to a median overall survival benefit. Raising awareness, decreasing the bowel cancer screening age, and prospective studies could improve EOCRC clinical outcomes.

## Introduction

Colorectal cancer (CRC) is the fourth most common cancer and the second most common cause of cancer death in the United Kingdom. The incidence of CRC in individuals over 50 years has remained stable or declined since the early 1990s.^1^ This observation is mainly due to the dissemination of bowel screening program, colon polypectomies as well as the increased public awareness and optimization of modifiable behaviors such as smoking reduction.^2,3^ On the contrary, there has been a significant increase in CRC cases in patients under 50 years, defined as early-onset CRC (EOCRC), a phenomenon that needs urgent research and intervention before it becomes a public health emergency.^1^ Numerous epidemiological studies conducted in Europe, Asia, and America have shown a similar trend not only in EOCRC but in a wide range of cancer types.^4-8^

Interestingly, most EOCRC cases are sporadic.^2,9^ People with a family history or hereditary syndromes predisposing them to CRC, such as Lynch syndrome or familial adenomatous polyposis, account in total for approximately 40% of EOCRC cases.^10-12^ Epidemiological studies have shown an association with obesity and type 2 diabetes mellitus in sporadic EOCRC.^10,13-16^ However, the role of smoking and alcohol consumption remain unclear.^17-21^ In addition, because of the alarming increase in the number of cases of EOCRC, the identification of other potentially modifiable risk factors has been the focus of research. A focused review of the commonest risk factors reported in the literature is presented in Table 1.

**Table 1.** Potentially modifiable risk factors for EOCRC, including the relevant rationale and available evidence.

Modifiable risk factors for EOCRC			
Risk factor	Rationale	Author/study type/study population	Findings
Diet-rich in red and processed meat	Growth-promoting dietary components lead to epithelial damage, proliferation, and DNA damage.^60-62^	Rosato et al^63^ Case-control study 329 CRC cases/1361 controls	Higher processed meat intake increased EOCRC risk, but there was no statistical significance for red meat and EOCRC.
		Chang et al^64^ Case-control study 175 CRC cases /253 controls	No statistically significant association between red or processed meat and EOCRC.
		Archambault et al^65^ Meta-analysis of 13 population-based studies 3767 EOCRC cases/4049 controls 23437 LOCRC cases/35 311 controls	Significant association between high red meat intake and EOCRC. No statistical association with processed meat.
Sugar-sweetened beverages (SSB)	Metabolic conditions are already a plausible risk factor of EOCRC. Increased intake of SSBs is linked with metabolic conditions and thus investigated as a link with EOCRC.^66^	Hur et al^66^ Prospective cohort study 95 464 females	Demonstrated that higher SSBs intake in adolescence and adulthood is associated with a higher risk of EOCRC among women.
		Chang et al^64^ Case-control study 175 CRC cases / 253 controls	Increased SSBs consumption may be associated with an increased EOCRC risk.
High vegetable/fruit intake	Associated with a higher abundance of the gut microbiome, Prevotella is believed to be associated with a lower risk of EOCRC.^67^	Chang et al^64^ Case-control study 175 CRC cases /253 controls	Greater vegetable intake showed a protective effect against EOCRC. No association between fruit intake and EOCRC.
		Rosato et al^63^ Case-control study 329 CRC cases/1361 controls	Greater vegetable intake showed a protective effect against EOCRC, as did a higher citrus fruit intake.
		Archambault et al^65^ Meta-analysis of 13 population-based studies 3767 EOCRC cases/4049 controls 23 437 LOCRC cases/35 311 controls	No statistical association among fruit, vegetable, and EOCRC risk.
Higher fiber intake	Thought to have a protective effect in later-onset CRC due to their role in increasing *Lactobacillus* spp. and butyrate-producing bacteria.^68,69^	Chang et al^64^ Case-control study 175 CRC cases/ 253 controls	No association between higher fiber intake and EOCRC.
		Chen et al^70^ Ecological analysis using sample sizes from the NHANES III survey (*n* = 5354) and continuous NHANES survey (*n* = 1574).	No association between higher fiber intake and EOCRC.
		Archambault et al^65^ Meta-analysis of 13 population-based studies 3767 EOCRC cases/4049 controls 23 437 LOCRC cases/35 311 controls	No association between higher fiber intake and EOCRC.
Vitamin D	Calcitriol inhibits proliferation and promotes epithelial differentiation of CDR-expressing human colonic carcinoma cell lines.^71^	Kim et al^72^ Prospective cohort study 94205 females from 1991 to 2015 111 EOCRC cases	Greater Vitamin D intake showed a protective effect against EOCRC in younger women.
		Hua et al.^73^ Systematic review/ meta-analysis *n* = 114112	Greater Vitamin D intake showed a protective effect against EOCRC.
Antibiotic exposure	Likely to impact the gut microbiota and lead to gut dysbiosis.^74^	McDowell et al.^75^ Case-control study 7903 CCR cases/445 EOCRC cases/30418 controls	Antibiotic exposure was associated with EOCRC.
		Nguyen et al.^76^ Case-control study 54 804 CRC cases/2557 EOCRC cases /261 089 controls	No association between antibiotic exposure and EOCRC.
Sedentary time	Sedentary time or lack of physical activity promotes gut dysbiosis.^77,78^	Chang et al.^64^ Case-control study 175 CRC cases/253 controls	Increased sedentary time is associated with a statistically significant increase in EOCRC risk.
		Nguyen et al^54^ Prospective cohort study 89 278 females	Increased sedentary time is associated with a statistically significant increase in EOCRC risk.
		Gu et al.^79^ Meta-analysis 32 843 EOCRC cases	Increased sedentary time is associated with a statistically significant increase in EOCRC risk.
		Archambault et al.^65^ Meta-analysis of 13 population-based studies 3767 EOCRC cases / 4049 controls 23 437 LOCRC cases / 35 311 controls	No association between increased sedentary time and EOCRC.
		Rosato et al.^63^ Case-control study 329 CRC cases/1361 controls	No association between increased sedentary time and EOCRC.

Abbreviations: CRC, colorectal cancer; EOCRC, early onset CRC; LOCRC, Late Onset CRC.

EOCRC is currently considered a heterogeneous disease with distinct molecular characteristics; however, the literature evaluating the molecular profile of EOCRC is currently limited. Studies show that younger patients tend to have more aggressive clinicopathological features at presentation when compared to their older counterparts.^22,23^ Younger patients are more likely to have advanced-stage disease at diagnosis, positive lymph nodes at presentation, and poorly differentiated or undifferentiated tumors.^5,22,24,25^ Finally, EOCRCs are associated with primarily left-sided tumors (74%-81.7%), with the most frequent being rectal and rectosigmoid primaries.^26,27^

There are currently no distinct guidelines for the treatment of EOCRC. Interestingly, and compared to late-onset colorectal cancer (LOCRC), patients with EOCRC are more likely to be treated more aggressively, irrespective of the stage of their cancer.^10^ Data on survival is conflicting, with some studies suggesting a less favorable outcome for younger patients,^28-30^ whereas others report better survival outcomes for patients with EOCRC.^31-34^

In 2021, the National Bowel Cancer Audit (NBOCA) published a report demonstrating a 0.6% increase in EOCRC cases in England over 4 years.^35^ The publication, which also commented on patient, tumor, and clinical characteristics as well as treatment modalities and cancer-specific survival, identified several areas of development. Evidently, more research is required to investigate and crystallize the risk factors, molecular drivers, treatment options, and prognosis of EOCRC. This study aims to characterize the clinical and pathological characteristics and the treatment outcomes of patients with EOCRC presenting at the Christie Hospital, a tertiary oncological center in the United Kingdom.

## Materials and Methods

### Eligibility criteria

This single-center retrospective study was conducted at a large comprehensive cancer center in Northwest England, UK (The Christie NHS Foundation Trust). Patients with a diagnosis of colorectal cancer under the age of 50 years between 1st January 2013 and 31st December 2021 were eligible for the study. The planned study follow-up ended on 28 February 2023. It is essential to mention that only patients with high-risk stage II and above EOCRC were likely to be referred to our cancer center and therefore included in this cohort. Patients were treated either with adjuvant systemic anticancer treatment post definite surgical intervention or received treatment for locally advanced, metastatic, or recurrent disease. This project was approved by the Quality Improvement and Clinical Audit (QICA) committee at the Christie NHS Number Trust. QICA number: 3510. Finally, patients referred for a second opinion and not treated at the Christie NHS Foundation Trust or with substantial data missing were excluded from the study (*n* = 25).

### Data collection

A list of patients with EOCRC was created by examining the electronic health records. The investigators then applied the above-mentioned eligibility criteria to select the study population.

The following data were collected for each patient: demographics, clinical presentation, risk factors, tumor characteristics, molecular profile, and treatment. Specifically, information was documented on the participant’s gender, age at diagnosis, date of diagnosis and, if applicable, date of death. For most patients, data were recorded on their presenting symptoms, including the nature of symptoms, onset, and duration before diagnosis. The location of the primary tumors of all the EOCRC cases was categorized into the following: ascending, transverse, descending, sigmoid colon, and rectum. Furthermore, information was recorded on the status of the primary tumor (resected vs in situ) and, if applicable, on the presence and sites of metastatic disease. The pathology American Joint Committee on Cancer tumor, node, metastasis (pTNM) staging system version 8 was documented for most patients. In some cases, when this was not available, the clinical staging (cTNM) was used instead.

In terms of risk factors, data were collected on smoking status, alcohol intake, obesity (defined as BMI ≥ 30 kg/m^2^), and family history of CRC. Each patient’s Eastern Cooperative Oncology Group (ECOG) performance status (PS) was noted at the initial consultation at the Christie. Data on comorbidities were collected based on the Adult Comorbidity Evaluation (ACE-27). This is a comorbidity index that considers 27 individual medical conditions for presence and severity while it grades them into 4 categories: none, mild, moderate, or severe.^36^ Based on the ailment with the highest score, the overall comorbidity score (OCS) is reported as 0 (none), 1 (mild), 2 (moderate), or 3 (severe).^36^ Large prospective registries have validated ACE-27 as a prognosticating tool for patients with cancer.^36,37^ Furthermore, we collected information on the pathology of the tumors including histopathological type and differentiation. Molecular parameters such as mismatch repair (MMR) status, RAS, BRAF, and PIK3CA were primarily reported. If available, the extended molecular profiling for somatic alterations was also collected.

Each patient’s treatment journey was recorded. This included information on surgical interventions, radiotherapy, and systemic anticancer therapy (SACT). The following data were obtained for each SACT regimen the patients received: setting (adjuvant, neoadjuvant, and palliative), line of treatment, cycles received, and tumor response if appropriate. Overall survival (OS) was calculated using the time between diagnosis and death from all causes or study follow-up ends. Moreover, progression-free survival (PFS) was defined as the time between SACT commencement and disease progression or death. Finally, the collected data were compared to the existing literature and the NBOCA report on EOCRC. As this report mainly studied patients with metastatic EOCRC, the comparison was focused on our subgroup of patients with de novo metastatic disease.^35^

### Statistical analysis

For all statistical analysis, the IBM SPSS version 25.0 was used. All the prementioned data categories underwent descriptive statistics. This included measures of frequency (counts, percentages), central tendency (mean and median), and variability (standard deviation (SD)). The independent *t* test and Mann-Whitney tests were used for the univariable analysis of continuous variables. Meanwhile, the Chi-square test was used for categorical variables. Kaplan-Meier curves were used to evaluate the OS and PFS while the log-rank test was applied for statistical comparison.

## Results

### Demographics and clinical data

The study included 203 patients diagnosed with EOCRC between January 1, 2013 and December 31, 2021. The population’s demographics are summarized in Table 2.

**Table 2.** Demographics, clinical data, and risk factors of the EOCRC patients.

Characteristics of patients with EOCRC	
Age, years	
Median, range Mean (male vs female) Mean (CRC vs no CRC family history)	42, 27-49 Male: 42.1, female: 41.1 (*P* = .18) CRC: 42.1, no CRC: 41.3 (*P* = .32)
Sex, *n* (%)	
Male	105 (51.7%)
Female	98 (48.3%)
Patients per year, *n* (%)	
2013-2015	9 (4.4%)
2016-2017	43 (21.2%)
2018-2019	33 (16.3%)
2020-2021	118 (58.1%)
Smoking status, *n* (%)	
Never	105 (51.7%)
Ex-smoker	47 (23.2%)
Current smoker	29 (14.3%)
Not known	22 (10.8%)
Alcohol consumption, *n* (%)	
<10 units	134 (66%)
≥10 units	47 (23.2%)
Not known	22 (10.8%)
BMI, *n* (%)	
<30 kg/m^2^	118 (58.1%)
≥30 kg/m^2^	44 (21.7%)
Not known	41 (20.2%)
Performance status, *n* (%)	
0	108 (53.2%)
1	74 (36.5%)
2	13 (6.4%)
3	2 (1.0%)
Not known	6 (3.0%)
Overall comorbidity score (OCS), %	
0-1	95.6%
≥2	4.4%
Predisposition, *n* (%)	
Sporadic cases	142 (70%)
Family history of CRC	55 (27%)
Hereditary syndromes	6 (3%)
Presenting symptoms, *n* (%)	
Altered bowel habit	90 (44.3%)
Abdominal pain	82 (40.4%)
Rectal bleeding	80 (39.4%)
Fatigue	26 (12.8%)
Weight loss	25 (12.3%)
Nausea and vomiting	10 (4.9%)
Rectal pain	6 (3%)
Duration of symptoms, months	
Median (range)	3 months (0-23)
0-3	99 patient (48.8%)
4-6	51 patients (25.1%)
≥7	40 patients (19.7%)
Not known	13 patients (6.4%)
Location of primary tumor, *n* (%)	
Rectal	74 (36.5%)
Sigmoid	59 (29.1%)
Ascending colon	35 (17.2%)
Transverse colon	24 (11.8%)
Descending colon	11 (5.4%)
Right- vs left-sided primary tumors, *n* (%)	
Left	144 (70.9%)
Right	59 (29.1%)
Stage at diagnosis, *n* (%)	
I	0 (0%)^a^
II	19 (9.4%) Right-sided: 7, left-sided: 12
III	77 (37.9%) Right-sided: 22, left-sided: 55
IV	107 (52.7%) Right-sided: 30, left-sided: 77
Sites of metastasis at referral, *n* (%)	
Liver	85 (79.4%)
Lung	30 (28%)
Omentum/peritoneum	21 (19.6%)
Ovary	4 (3.7%)
Bone	2 (1.9%)

^a^Only patients with high-risk stage II and above EOCRC were likely to be referred to the cancer center

### Pathology and molecular profile

The pathological and molecular features of our EOCRC population are summarized in Table 3.

**Table 3.** Pathological and molecular features of EOCRC patients, including histology, differentiation, MMR, KRAS, NRAS, BRAF, and PIK3CA status.

Pathology and molecular profile of patients with EOCRC	
Histology, *n* (%)	
Adenocarcinoma	182 (89.7%)
Mucinous adenocarcinoma	17 (8.4%)
Signet ring adenocarcinoma	3 (1.5%)
Not known	1 (0.5%)
Differentiation, *n* (%)	
Well	4 (2.0%)
Moderate/poor	175 (86.2%)/23 (11.3%)
Not known	24 (11.8%)
MMR status, *n* (%) ^a^	
Assessed	160 (78.8%)
Proficient	140 (87.5%)
Deficient	20 (12.5%) in total In stage II: 4 (22%) In stage III: 11 (20%) In stage IV: 5 (6%) (*P* = 0.015)
MMR status Proficient Deficient	Primary tumor location Right: 43 (31%), left: 97 (69%) Right: 11 (55%), left: 9 (45%) (*P* = 0.032)
KRAS status, *n* (%)	
Assessed	153 (75.4%)
Wild type	88 (57.5%)
Mutated	65 (42.5%)
	G12D: 24 (36.9%)
	G12V: 13 (20.0%)
	Unknown: 8 (12.3%)
	G12S: 5 (7.7%)
	G12C: 4 (6.2%)
	G13D: 4 (6.2%)
	Q61H: 2 (3.1%)
	Q61L: 2 (3.1%)
	G12F: 1 (1.5%)
	G12R: 1 (1.5%)
	G12A: 1 (1.5%)
KRAS status Wild type Mutated	Primary tumor location Right: 28 (32%), left: 60 (68%) Right: 23 (35%), left: 42 (65%) (*P* = 0.64)
NRAS status, *n* (%)	
Assessed	144 (70.9%)
Wild type	138 (95.8%)
Mutated	6 (4.2%)
NRAS status Wild type Mutated	Primary tumor location Right: 45 (33%), left: 93 (67%) Right: 2 (33%), left: 4 (67%) (*P* = .97)
BRAF status, *n* (%)	
Assessed	144 (70.9%)
Wild type	124 (86.1%)
Mutated	20 (13.9%) V600E: 16 (80.0%)
	D594G: 2 (10%)
	V600D: 1 (5%)
	G466G: 1 (5%)
BRAF status Wild type Mutated	Primary tumor location Right: 35 (28%), left: 89 (72%) Right: 12 (60%), left: 8 (40%) (*P* = .005)
PIK3CA status, *n* (%)	
Assessed	130 (64%)
Wild type	110 (84.6%)
Mutated	20 (15.4%)
PIK3CA status Wild type Mutated	Primary tumor location Right: 35 (32%), left: 75 (68%) Right: 9 (45%), left: 11 (55%) (*P* = .25)

^a^MMR testing became mandatory for all patients with CRC in 2017.

Abbreviations: EOCRC, early-onset colorectal cancer; MMR, mismatch repair.

### Treatment

As far as treatment modalities are concerned, the surgical interventions offered in this EOCRC population are summarized in Table 4. These interventions included primary tumor resection, de-functioning stomas, and metastasectomies. The administration of radical and palliative radiotherapy is also reported in Table 4.

**Table 4.** Treatment modalities including surgery, radiotherapy, and SACT in this EOCRC population stratified by stage.

Treatment modalities in EOCRC				
		Stage II	Stage III	Stage IV
Surgical intervention	Primary resection	15 (78.9%)	65 (84.4%)	43 (40.2%)
	Defunctioning	3 (15.8%)	14 (18.2%)	24 (22.4%)
	Metastasectomy	0 (0%)	3 (3.9%)^a^	25 (23.4%)
	Liver resection	0 (0%)	1 (1.3%)^a^	22 (20.6%)
Radiotherapy	Radical	4 (21.1%)	23 (29.9%)	13 (12.1%)
	Palliative	0 (0%)	2 (2.6%)^a^	16 (15%)
SACT	Neoadjuvant	6 (31.6%)	30 (39.0%)	29 (27.1%)
	Adjuvant	12 (63.2%)	45 (58.4%)	17 (15.9%)
Relapse		4 (21.1%)	30 (39.0%)	N/A
Palliative SACT (de novo/relapsed metastatic setting)	1st line	4 (3.1%)	30 (23.1%)	96 (73.8%)
	2nd line	1 (1.1%)	20 (23.0%)	66 (75.9%)
	3rd line	1 (2.4%)	6 (14.3%)	35 (83.3%)

^a^At relapsed metastatic setting.

Abbreviations: EOCRC, early-onset colorectal cancer; SACT, systemic anticancer treatment.

In terms of SACT, the use of neoadjuvant, adjuvant, and palliative chemotherapy by stage, where applicable, is presented in Table 4. Doublet regimens with combinations of either capecitabine or 5-FU with oxaliplatin were administered for most patients in the adjuvant and neoadjuvant setting (*n* = 103, 83.0%). Monotherapy with capecitabine was given neoadjuvantly with concurrent radiotherapy in 10 patients (8.1%) while adjuvant capecitabine was the treatment of choice in 11 patients (8.9%) mainly due to T3N0 (stage II) tumors, patient’s decision and during the COVID-19 pandemic. In first-line palliative chemotherapy, the combination of oxaliplatin or irinotecan with either 5-FU or capecitabine was the backbones of SACT, while anti-EGFR antibodies (panitumumab, cetuximab) were administered in EOCRCs with wild-type RAS. In 2 patients, the regimen was escalated to combination of oxaliplatin, irinotecan, and 5-FU (FOLFOXIRI). The median number of cycles administered was 11 (21 weeks of treatment). Disease control in the de-novo and relapsed metastatic setting was observed in 53.1% (stable disease (SD) in 14.6%, partial response in 36.2%, and complete response (CR) in 2.3% of the cases). In one-third of the cases (33.9%), the disease progressed after first-line treatment. Finally, mixed responses were seen in 3.8% of the cohort (9.2% with incomplete response information).

Second-line SACT was offered in 67% of the patients who progressed on first-line treatment. Combination of 5-FU or capecitabine with either oxaliplatin or irinotecan (whichever was not selected in the first line) was the commonest SACT regimens, followed by encorafenib/cetuximab for patients with BRAF V600E mutations. The median number of cycles was 6 (12 weeks of treatment); disappointingly, more than half of the patients (52.9%) experienced PD on chemotherapy. Third-line SACT, mainly with trifluridine and tipiracil, was given in 42 patients (32.0%). The median number of cycles was 4 (11 weeks of treatment); the best response was PD in 78% of the population. Finally, immunotherapy with single-agent nivolumab was offered in 4 patients with EOCRC and MMR deficiency in the second- and third-line setting. The treatment regimens across all SACT lines are summarized in Table 5.

**Table 5.** Treatment regimens offered as 1st, 2nd, and 3rd line as well as the associated median PFS in patients diagnosed with EOCRC.

First-line SACT	Patients	Second-line SACT	Patients	Third-line SACT	Patients
FOLFIRI	35	FOLFIRI	33	Trifluridine/tipiracil	16
FOLFOX	31	FOLFOX	22	FOLFIRI	7
FOLFOX + panitumumab	15	Encorafenib + cetuximab	6	FOLFOX	7
FOLFIRI + cetuximab	14	Trifluridine/tipiracil	6	Trial	3
FOLFOX + cetuximab	7	Trial	5	Nivolumab	2
Capecitabine + oxaliplatin	7	Capecitabine + irinotecan	3	FOLFOX + bevacizumab	1
Irinotecan	5	FOLFIRI + bevacizumab	2	Irinotecan	1
FOLFIRI + panitumumab	4	Nivolumab	2	Capecitabine + irinotecan	1
FOLFOXIRI	2	FOLFIRI + aflibercept	1	FOLFIRI + bevacizumab	1
Encorafenib + cetuximab	2	FOLFIRI + panitumumab	1	Encorafenib + cetuximab	1
Irinotecan + cetuximab	2	FOLFIRI + cetuximab	1	Capecitabine + bevacizumab	1
Capecitabine	2	Irinotecan	1	Regorafenib	1
Oxaliplatin + raltitrexed	1	Irinotecan + cetuximab	1		
FOLFOX + bevacizumab	1	FOLFOXIRI	1		
Capecitabine + irinotecan	1	Capecitabine	1		
Raltitrexed	1	Oxaliplatin + raltitrexed	1		
Total	130		87		42
					
Median PFS (months)	6		3		2

Abbreviations: EOCRC, early-onset colorectal cancer; SACT, systemic anticancer treatment; FOLFIRI, Folinic acid; fluorouracil and Irinotecan; FOLFOX, folinic acid; fluorouracil and oxaliplatin; FOLFOXIRI, folinic acid; fluorouracil; oxaliplatin and irinotecan.

### Prognosis and survival analysis

**Figure 1.**
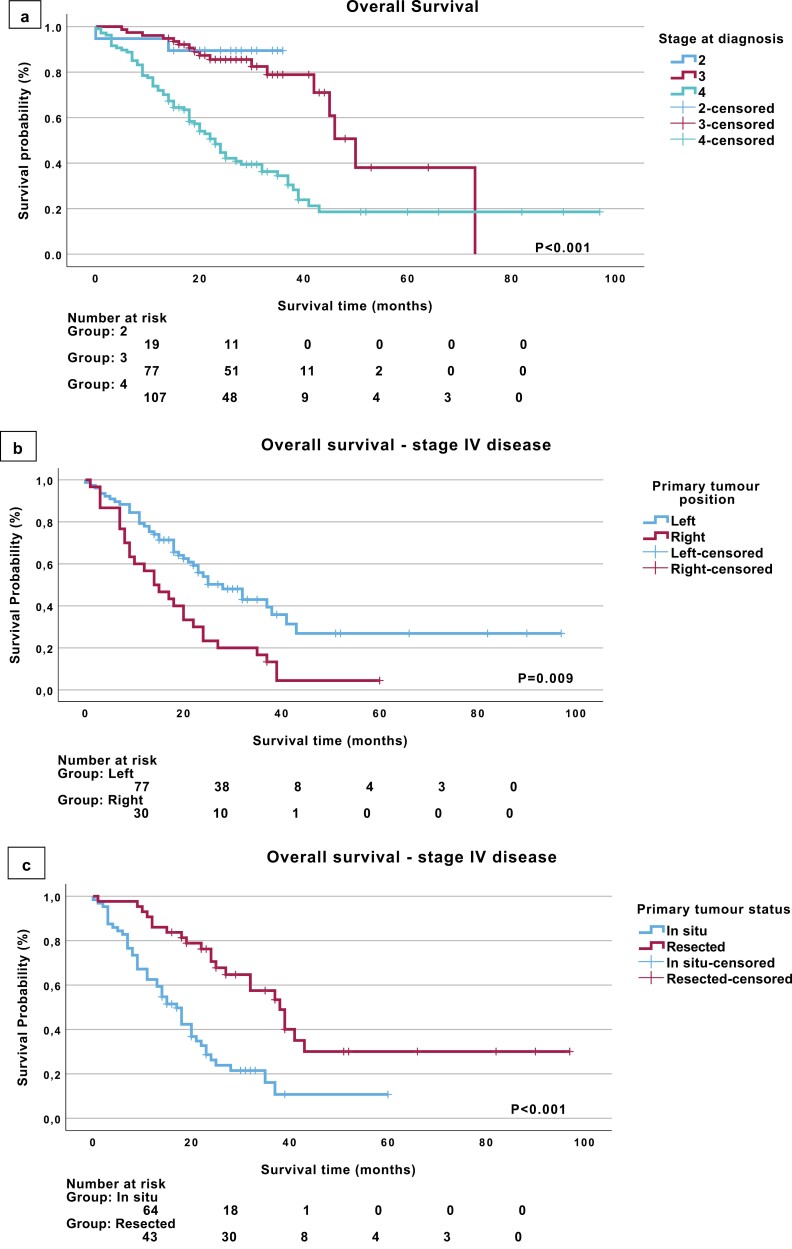
(a) OS comparison between patients with stages II, III, and IV EOCRC, (b) OS comparison between right- and left-sided primary tumors in patients with de-novo stage IV EOCRC, (c) OS comparison between resected and in situ primary tumors in patients with de novo stage IV EOCRC. Abbreviations: EOCRC, Early Onset Colorectal Cancer; OS, overall survival.

At the end of the planned study follow-up (February 28, 2023), the median follow-up time was 22 months (range 1-97 months). During this period, we documented 89 deaths (43.8%) in this cohort of patients with EOCRC, while there were 2, 17, and 70 reported deaths for stages II, III, and IV cancers, respectively. The median OS for the entire cohort was 38 months (95% CI, 32.86-43.14), and the OS at 12 months and 3 years was 83.3% and 52.6%, respectively. Patients with stage IV disease at diagnosis experienced the worst median OS of 23 months (95% CI, 19.11-26.88) vs 50 months (95% CI, 43.52-56.48) for patients with stage III disease. The median OS for stage II EOCRCs was not reached (Figure 1a).

We studied the effect of multiple factors on OS of the whole population including gender, age group, duration of symptoms, smoking, alcohol, obesity, and family history of CRC. There were no statistically significant OS differences in univariate analysis. Similarly, in the stratified analysis by disease stage, there were no significant survival differences.

We assessed the effect of primary tumor location (right vs left) on OS. In the whole population, there was a trend toward improved median OS for left-sided (41 months, 95% CI, 32.47-49.53) vs right-sided (35 months, 95% CI, 20.51-49.49) primary tumors, although not statistically significant (*P* = .066). In subgroup analysis, there was no statistically significant difference identified for patients with stages II and III disease. However, in the de-novo metastatic population, right-sided primary tumors were associated with worse median OS of 14 months (95% CI 7.29-20.71) compared to 28 months (95% CI 19.37-36.63) for left-sided primaries (*P* = .009; Figure 1b).

The effect of primary tumor status was also studied, and we showed that patients with resected tumors had a median OS of 45 months (95% CI, 39.38-50.62) compared to 18 months for patients with in situ tumors (95% CI, 14.54-21.46) (*P* < .001). As expected, in the non-metastatic setting (stages II, III), the median OS was 50 months (95% CI, 40.75-59.25) for patients with resected primaries compared to 22 months (95% CI, 14.48-29.52) for patients with in situ primaries. Interestingly, in stage IV disease, primary tumor resection was associated with a median OS benefit of 21 months (38 vs 17 months, *P* < .001) compared to in situ tumors (Figure 1c).

We reported earlier that neoadjuvant chemotherapy was offered in 65 patients across all stages. In patients with stages II and III disease, there was no correlation between neoadjuvant chemotherapy and OS. However, there was a clear survival benefit of 21 months for patients with stage IV EOCRC who received neoadjuvant chemotherapy (median OS 39 months, 95% CI, 25.00-43.00) vs those that they did not (median OS 18 months, 95% CI, 14.00-23.00) (*P* = .0002). Finally, we reviewed the PFS post first-, second-, and third-line palliative chemotherapy in patients with EOCRC. Median PFS post first-line SACT was reported as 6 months (95% CI, 4.85-7.15); after the second-line chemotherapy, median PFS dropped to 3 months (95% CI, 2.56-3.44). Similarly, median PFS after third-line SACT was even lower at 2 months (95% CI, 1.60-2.40).

## Discussion

The incidence and mortality rates of EOCRC have been rising since the 1970s, especially in the subgroup of 40-49 years.^5,38^ To the best of our knowledge, this is the first UK-based study collecting real-world data on EOCRC from a single tertiary oncology center over 9 years.

The patients in the current study represented approximately 10% of all patients with CRC treated in our center between 2013 and 2021. Interestingly, the NBOCA on EOCRC in England between 2013 and 2018 showed an increasing incidence of EOCRC from 2013-2014 to 2017-2018, with a percentage of 5.2% and 5.8%, respectively. In our cohort, we reported a significant increase in EOCRC cases between 2018-2019 (*n* = 33) and 2020-2021 (*n* = 118). This significant increase can be mainly attributed to external referrals for clinical trials and demonstrates a largely unmet need in these young patients. Finally, a relatively equal distribution between males and females was reported in NBOCA, and we confirmed that in our study.^35^

Several studies have correlated the presence of advanced disease at diagnosis of EOCRC with prolonged symptom duration and delayed diagnosis.^10,39^ However, conflicting evidence has also been found, and one study showed that patients with non-advanced EOCRC had a longer duration of presenting symptoms compared to patients with advanced EOCRC.^40^ In our population, we demonstrated that almost 74% of the patients had symptoms for ≤6 months, while 25% had a duration of symptoms between 3 and 6 months. Interestingly, there was no statistically significant relationship between the duration of symptoms (≤6 months vs >6 months) and the presence of metastatic disease at diagnosis (*P* = .75), while the median OS was not adversely affected either. However, one in 5 young patients might experience symptoms for more than 6 months, and it is important to highlight the growing problem of EOCRC through awareness campaigns and targeted education in primary care.

Left-sided primary tumors are more frequent in patients with EOCRC, which was confirmed in our study, with left-sided tumors accounting for approximately 70%.^2,9^ Possible reasons include that EOCRC-associated risk factors (eg, Western diet) predominantly cause left-sided cancers. In contrast, factors associated with LOCRC (eg, smoking) tend to lead to right-sided primaries.^41^ Interestingly, we also demonstrated that right-sided primary tumors were strongly associated with a median OS disadvantage of 14 months compared to left-sided primary tumors in patients with de novo stage IV EOCRC (*P* = .009). Other studies have also showed increased mortality for right-sided CRCs although these mainly included older patients.^42,43^ Nevertheless, Sanford et al, in their study of EOCRC reported a higher mortality for younger patients (18-29 years) with left-sided colon cancers, while for the older groups aged 30-39 and 40-49 years, mortality was similar between right and left-sided colon cancers.^41^

The majority of patients in our study were fit and well, with a reported ECOG PS of 0-1 in 89.7% of the patients and a good overall comorbidity score (OCS), as it would be expected in a young cohort of patients and is consistent with previous reports.^9,44^ Common risk factors for CRC were infrequent in our cohort, as 51.7% of our patients were never smokers, 66% consumed alcohol within the recommended limit, and 58.1% were non-obese. It has been suggested in the literature that factors such as dietary preferences, antibiotics, and prenatal to early adulthood exposures can lead to gut inflammation and dysbiosis. These elements have been correlated with EOCRC.^38,39^ Prospective studies will need to collect real-life data on these factors.

In terms of pathological factors, the frequency of mucinous adenocarcinoma and signet ring adenocarcinoma was similar to that reported in the literature. At the same time, we reported a lower number of poorly differentiated tumors (11.3% vs 27.9%).^45^ This observation could be related to the fact that nearly 57% of patients in our study were aged >40 years, and Vuik et al in their study suggested that the frequency of poorly differentiated tumors decreases as the age of diagnosis increases in patients with EOCRC (16.6% in the age group 40-49 years).^46^ Furthermore, chromosomal instability (CIN) was the dominant molecular pathway in our cohort. CIN is characterized by microsatellite stability, left-sided primary tumors, as well as KRAS and PIK3CA mutations, and our findings were consistent with the current literature.^25,45^

Several studies have shown that patients with EOCRC are treated more aggressively than patients with LOCRC.^44,47,48^ In the de novo metastatic disease setting, the NBOCA reported that 91% of patients with EOCRC received at least one treatment modality, while this percentage dropped to 82% and 51% for the age groups 50-70 years and >70 years, respectively.^35^ In our de novo metastatic cohort, all patients received at least one cancer-related treatment. We described a similar number of primary resections (40.2% vs 41%) and a higher number of metastasectomies (23.4% vs 17%). Most of the latter included hepatectomies for colorectal liver metastases (CRLM). According to the institutional criteria, CRLMs are considered resectable when there is potential for complete resection (R0), preservation of 2 or more liver segments with viable vascular inflow/outflow and biliary drainage while future liver remnant is > 25%.^49^ Furthermore, any type of chemotherapy was offered in 98% of our population compared to 83% of the patients in NBOCA. As our center receives referrals for 2nd opinions and trials, our population will be slightly skewed to fit patients who are keen to pursue further treatment which may explain higher treatment rates. Finally, these percentages were considerably lower in the NBOCA age groups 50-70 years (71%) and >70 years (33%), a fact that the higher number of comorbidities and worse PS can explain.

Our survival analysis in the de novo metastatic subgroup showed a 2-year OS of 44.6% compared to 37.4% in the EOCRC group of the NBOCA.^35^ This finding can probably be attributed to the above mentioned higher rates of SACT and that patients were treated in a cancer center.^50^ Pooled analysis from 9 phase III clinical trials evaluating first-line SACT demonstrated a median OS of 15.8 and 16.6 months in EOCRC and LOCRC groups, respectively.^51^ Our single-center study in patients with EOCRC showed a median OS of 23 months. Moreover, in the same subgroup (stage IV), we demonstrated a median OS benefit of 21 months for patients with resected primary tumors. This finding has also been reported in a large observational study of 6708 patients with EOCRC, in which primary tumor resection was correlated with improved OS (HR 0.46, 95% CI, 0.43-0.49, *P* < .001).^52^

We acknowledge the limitations of this study. The retrospective nature limited our access to risk factors such as early life exposures, diet, and physical activity while an information bias cannot be ruled out on smoking status, alcohol intake, and family history of CRC. Furthermore, a selection bias cannot be excluded as the patients referred and reviewed at our center were diagnosed with high-risk stage II and above EOCRC and were fit enough for oncological management. Finally, our data comparison were restricted to the NBOCA and the current literature due to the absence of a control group of patients with LOCRC.

There are multiple possible causes for the rising incidence of EOCRC related to diet, lifestyle, and environment. It is very difficult to know which of the currently identified factors are most important when considering possible preventative strategies. Starting from primary prevention early in life, targeted interventions would include adopting healthier dietary habits (eg, higher fiber intake and reduced ultra-processed foods) and promoting physical activity, alongside addressing factors (eg, antibiotic exposure in infancy) that could lead to a reduction in childhood and adolescent obesity, which have been correlated with EOCRC.^53-55^ More specifically, antibiotics can directly affect the gut microbiota, leading to altered immune responses with increased inflammation that may promote carcinogenesis.^56^

When considering secondary prevention, improving adherence to early screening programs for individuals with a family history of CRC or hereditary syndromes is paramount, as this population almost comprise 40% of EOCRC. Of course, 60% of EOCRC cases remain sporadic, and growing evidence proposes that the CRC screening age should be lowered to <50 years. Almost 50% of patients with EOCRC are diagnosed in the USA between 45 and 49 years, and the American Cancer Society suggested commencing CRC screening at the age of 45 years.^10,57^ Raising awareness of the red flag symptoms among the general public and health care professionals is essential to speed up diagnosis and treatment.

As it has been reported in the literature, patients with ΕOCRC are overall treated more aggressively with the traditional anticancer modalities without improved outcomes^44,47,48^ The disappointing PFS in EOCRC creates the need for a change in the current approach. Comprehensive genomic profiling should be considered at the point of diagnosis to allow patients with EOCRC to be matched to an increasing number of approved targeted treatments or in the context of clinical trials. Furthermore, EOCRC has been identified as a priority area for research and there are now several large-scale funded research projects investigating the causes, risk factors, and interventions. An example is the team PROSPECT, funded by the CRUK Grand Challenges, which aims to identify the relationship between exposome and EOCRC as well as to develop strategies for EOCRC prevention.^58^

Finally, adopting a holistic approach to managing patients with EOCRC would be essential. The diagnosis of EOCRC undoubtedly carries a significant psychological, professional, financial, and family planning impact for these patients. Future work should focus on patients living with and beyond their EOCRC diagnosis by expanding the support and well-being services in both adult and teenage and young adults (< 25 years old) settings. In that direction, “Bowel Cancer UK,” the leading bowel cancer charity in the UK, launched the “Never Too Young” campaign in 2013 and the “Never Too Young” project in 2020. The aim has been to raise overall awareness, inform and support patients diagnosed with EOCRC, and suggest policy changes that would lead to early diagnosis and improve treatment and overall care of this group.^59^

## Conclusions

Our study and the broader literature have clearly shown that the burden of EOCRC has been increasing over recent years, and it is predicted that it will continue rising at least until 2030. EOCRC has distinct characteristics, including predominantly left-sided tumors, advanced disease at diagnosis, and aggressive pathology. Retrospective studies have suggested a correlation between EOCRC and exposure to modifiable risk factors (eg, diet, obesity, and antibiotics) during early prenatal to adolescent life; hence, adulthood risk factors have not been strongly correlated with this subgroup of CRC to date.^39^ The molecular drivers have not been fully established, and stratified treatment protocols have not been developed.

Undoubtedly, there is an urgent need to improve our knowledge of the underlying mechanisms leading to EOCRC. It is also crucial that prospective clinical trials focus more on EOCRC, and patients are encouraged to participate in these studies. Some measures, such as raising awareness of EOCRC and decreasing the bowel cancer screening age, could be deployed imminently, while others, such as prospective studies and targeted drug development, will require significantly more resources and time. Ultimately, the aim would be to improve EOCRC prevention and early diagnosis while offering more effective treatments and tailored support that will lead to better survival outcomes for this young population.

## Data Availability

The data underlying this article will be shared on reasonable request to the corresponding author.
